# Using Next-Generation Sequencing to Analyse the Diet of a Highly Endangered Land Snail (*Powelliphanta augusta*) Feeding on Endemic Earthworms

**DOI:** 10.1371/journal.pone.0075962

**Published:** 2013-09-27

**Authors:** Stéphane Boyer, Stephen D. Wratten, Andrew Holyoake, Jawad Abdelkrim, Robert H. Cruickshank

**Affiliations:** 1 Bio-Protection Research Centre, Lincoln University, Lincoln, New Zealand; 2 Department of Ecology, Faculty of Agriculture and Life Sciences, Lincoln University, Lincoln, New Zealand; 3 Centre for Reproduction and Genomics, Department of Anatomy & Structural Biology, University of Otago, Dunedin, New Zealand; 4 Unité Conservation des Espèces, Restauration et Suivi des Populations, UMR 7204, Muséum National d’Histoire Naturelle, Paris, France; Agriculture and Agri-Food Canada, Canada

## Abstract

Predation is often difficult to observe or quantify for species that are rare, very small, aquatic or nocturnal. The assessment of such species’ diet can be conducted using molecular methods that target prey DNA remaining in predators’ guts and faeces. These techniques do not require high taxonomic expertise, are applicable to soft-bodied prey and allow for identification at the species level. However, for generalist predators, the presence of mixed prey DNA in guts and faeces can be a major impediment as it requires development of specific primers for each potential prey species for standard (Sanger) sequencing. Therefore, next generation sequencing methods have recently been applied to such situations. In this study, we used 454-pyrosequencing to analyse the diet of *Powelliphantaaugusta* , a carnivorous landsnail endemic to New Zealand and critically endangered after most of its natural habitat has been lost to opencast mining. This species was suspected to feed mainly on earthworms. Although earthworm tissue was not detectable in snail faeces, earthworm DNA was still present in sufficient quantity to conduct molecular analyses. Based on faecal samples collected from 46 landsnails, our analysis provided a complete map of the earthworm-based diet of *P* *. augusta* . Predated species appear to be earthworms that live in the leaf litter or earthworms that come to the soil surface at night to feed on the leaf litter. This indicates that *P* *. augusta* may not be selective and probably predates any earthworm encountered in the leaf litter. These findings are crucial for selecting future translocation areas for this highly endangered species. The molecular diet analysis protocol used here is particularly appropriate to study the diet of generalist predators that feed on liquid or soft-bodied prey. Because it is non-harmful and non-disturbing for the studied animals, it is also applicable to any species of conservation interest.

## Introduction

### Molecular analysis of animal diets

The study of animal diets is of major importance in conservation biology and in biological control of pests (e.g. [1–6]). The classical approach to diet analysis often relies on the morphological examination of gut content or faeces. Remains such as plant leaves and seeds, insect cuticle, mammalian hairs and teeth can be retrieved and identified to species or higher taxa based on their morphology. However, this method often lacks precision at the species level particularly for predators that feed on soft-bodied prey such as molluscs or earthworms and predators that masticate their prey thoroughly such as bats [7]. It is also not applicable to species that feed on liquid (e.g. Hemiptera, spiders etc.).

Molecular techniques targeting prey DNA remaining in gut or faeces of predators [8] can be a useful alternative to morphological methods because (i) it allows identification at the species level providing that those species have been sequenced before, (ii) it is applicable to soft-bodied prey, and (iii) it is applicable to liquid feeders. These techniques are based on the use of polymerase chain reactions (PCR) on prey DNA remaining in predators’ guts or faeces.

Therefore, molecular methods have been recently applied to study the diet of various species for which feeding is difficult to observe or quantify. These include species that are very small [9], aquatic [10] nocturnal [7] or elusive [11].

One important limitation of these methods is the degradation of prey DNA through digestion. This leads to mainly small DNA fragments surviving digestion, so that recently eaten prey (in gut samples) are easier to detect than are prey eaten earlier (in faeces). However, in the case of endangered predator species it is not desirable to sacrifice individuals to study their diet. Therefore, from a conservation perspective, gut content samples can only be sourced from fresh, accidentally killed, individuals [e.g. [12]] while faecal samples can be much easier to obtain [e.g. [13]].

### Faecal molecular analyses

Despite its easy collection, the analysis of prey DNA from predator faeces faces several technical challenges [14,15].

(1) Because DNA is degraded during digestion, little prey DNA can be retrieved from the faeces and only small fragments of it remain [16,17]. Therefore, for optimal efficiency, the PCR primers must target a short DNA fragment. Reliable PCR results from digested DNA have been obtained using fragments of less than 300 base pairs [18–22]. Furthermore, mitochondrial and ribosomal DNA, are more likely to be retrieved than nuclear DNA after digestion due to high copy number per cell [8].

(2) Faeces may contain a mix of DNA from different prey species, the predator itself, gut bacteria, parasites and accidentally swallowed items. Therefore identification of the predated species requires specific primers [e.g. [8]]. For generalist predators, this method is very demanding, as specific primers need to be designed and tested for each potential prey species [23]. It follows that DNA of unsuspected prey would remain unnoticed. Group-specific primers can be used to focus on a particular group of closely related species. However, when DNA from several species of prey is present in one faecal sample, group-specific primers and conventional Sanger sequencing are not compatible [e.g. [15]] as the latter requires there to be thousands of copies of the same DNA fragment to produce a consensus sequence.

**Figure 1:**
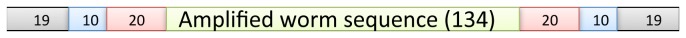
Modified primer used for PCR amplification prior to 454-pyrosequencing. Pyrosequencing fusion primers were as recommended by the manufacturer (Roche), molecular tags were as recommended by Parameswaran et al. (2007) [45] and New Zealand endemic earthworm group-specific primers were as described in Boyer et al. (2011) [15].

(3) As a consequence, once prey DNA has been amplified from predator faeces, post-PCR processing such as high-resolution melting (HRM) [e.g. [24]], single-strand conformational polymorphism (SSCP) [25], restriction fragment length polymorphism (RFLP) [e.g. [26]], denaturing gradient gel electrophoresis (DGGE) (e.g. [27]), or subcloning (e.g. [16,28]) is required to separate DNA from different prey species. Identification of predated species also requires prior establishment of the behaviour of each of the potential species’ DNA in the chosen post-PCR process. This can be time-consuming and requires much laboratory work, which increases the risk of errors and becomes expensive if a large number of samples need to be analysed.

Next-generation sequencing (NGS) technologies such as 454-pyrosequencing offer a much simpler alternative by detecting and sequencing many thousands of DNA fragments simultaneously from mixed samples [29–31]. Pyrosequencing has been used largely for whole genome sequencing and the sequencing of environmental DNA samples [31,32]. Recently it has been successfully applied to diet analysis both under controlled feeding conditions [33] and for wild animals [13].

In this study we used 454-pyrosequencing to investigate the earthworm-based diet of *Powelliphantaaugusta* Walker Trewick & Barker [34] (Mollusca: Pulmonata: Rhytididae), a highly endangered species of land snail endemic to New Zealand [35]. The original habitat of *P* *. augusta* is Mount Augustus on the western scarp of the Stockton Plateau (West Coast of New Zealand’s South Island) most of which was lost to open-cast coal mining at the Stockton mine in 2007. Following a decision from the environmental court of New Zealand, a systematic collection campaign was launched in 2006 by the mining company Solid Energy New Zealand Limited in conjunction with the New Zealand Department of Conservation to conserve the species and allow the mining operations to continue. Hand-collected individuals (6140 snails of various age and 1116 eggs) were either relocated to adjacent undisturbed areas outside the planned mine footprint or kept and cultured in captivity for re-introduction once the original site has been rehabilitated after coal extraction (c. 10 years). Although previous studies have shown that *P* *. augusta* almost exclusively feeds on earthworms [15], the identity of the predated species as well as their relative contributions remains unknown. This study aims at providing a detailed analysis of the snail’s diet to inform the conservation programme and ensure long-term survival of relocated and captive populations.

## Materials and Methods

### Ethics statement

*P* *. augusta* snails collected in the field were placed in individual clean plastic containers for 48 hours in appropriate moisture and temperature conditions. Only the faecal strings produced during that time were retained for DNA analysis. Faeces were naturally excreted. Snails were returned to the wild, unharmed, after the two day holding period.

Animal handling and sampling methods were conducted according to relevant national and international guidelines. All necessary permits were obtained from the New Zealand Department of Conservation (permit numbers: WC-19030-FAU and WC-25283-FAU). These permits were issued under section 53 of the Wildlife Act 1953.

### Land snail faecal samples

Molecular analyses were conducted on 46 snail faecal strings obtained from 46 different individuals collected from the field in November 2006 and May 2007. Because earthworms’ soft bodies leave no recognisable tissue that could be analysed individually after digestion by the snails [15], DNA extractions were performed on a bulk sample of each snail faecal string. The Qiagen DNeasy® blood and tissue kit was used to extract DNA from snail faeces. Snail diet was compared in relation to snail age, with the aim of detecting potential ontogenic shifts. Snail age was estimated by the maximum diameter of their shell; four categories were distinguished: hatchlings < 13 mm; juveniles < 20 mm; sub-adults < 32 mm; and adults ≥ 32 mm.

### Earthworm DNA library, mini-barcode selection and molecular tags

Previous morphological and molecular analysis of faecal samples revealed that the diet of *P* *. augusta* is mainly based on endemic New Zealand earthworms (Oligochaeta: Acanthodrilidae, Megascolecidae) [15]. An inventory of earthworms on the Stockton plateau was made from 2008 to 2010 and involved the collection of more than 1,500 individuals [36] from the remainder of the original range of *P* *. augusta* as well as similar habitat in surrounding areas [37]. Based on this inventory, a DNA library for all potentially predated earthworm species was built using DNA sequences from 139 earthworm specimens, selected to maximize taxonomic representation.

The mitochondrial 16S rDNA gene was chosen for species delineation, as this molecular marker is suitable for earthworm taxonomy, both at genus and species level [38,39].

The obtained DNA library included 15 clades separated by genetic differences greater than 10%. Based on this divergence, those clades were recognised as different species [36]. Most of these species are yet to be described; however, four of them have been identified based on previous taxonomic descriptions [40,41]. These four are: *Deinodrilus* gorgon Blakemore (referred to as species 1 in this study); *Eudinodriloides* *forsteri* Lee (referred to as species 3 in this study); *Octochaetuskenleei* Blakemore (referred to as species 7 in this study); and *Maoridrilus* *felix* Blakemore (referred to as species 9 in this study).

The sliding window function available in the R package SPIDER (Species Identity and Evolution in R) [42] was used to determine the shortest DNA fragment or ‘mini-barcode’ that displayed enough variability to accurately identify all earthworm species occurring in the snails’ geographic range [43]. The selected mini-barcode was a 134 bp fragment starting at position 11922 of the published *Lumbricus terrestris* Linnaeus mitochondrial genome sequence [44]. Group-specific primers designed to amplify this mini-barcode in New Zealand endemic earthworms [43] were: primer A (nz worm 16S int F) 5′ -AATTMGGTTGGGGCGACSHW-3′ ; primer B (nz worm 16S int R) 5′-AACATCGAGGTGCCAAWCCC-3. These were modified for 454-pyrosequencing with fusion primers added at both ends according to the manufacturer’s recommendation for the *Roche Genome Sequencer FLX System* and molecular identifiers (MID) of 10 base pairs [45] included between the fusion primers and the group specific primers (Figure 1). The MID ensure that the origin of each amplicon could be traced, i.e., which snail faecal string they were derived from, thus allowing the analysis of individual snail diet and comparison between snail age classes. Statistical analyses (ANOVA, permutational multivariate analysis of variance and *F*-test) were performed in R [46] using the packages *stats* and *vegan*.

### Nested PCR protocol

To optimise the amplification of earthworm DNA from snail faeces, a nested PCR approach was used. This consisted of a first PCR using a pair of universal invertebrate primers (LR-J-12887 and LR-N-13398 [47]), which amplify a ~500 base pair fragment of the 16S mitochondrial gene, followed by a 10x dilution of the PCR products and a second (nested) PCR using the modified group-specific primers described above (Figure 1), which amplify a 134 base pair fragment. Each PCR product contained a unique molecular tag corresponding to the land snail faecal string from which it came.

For both PCRs, the 25µl reactions contained 5µl Qiagen Q solution, 2.5µl 10X buffer (Invitrogen), 2.5µl dNTPs [2.5mM each], 1µl MgCl_2_ [25mM], 1µl Bovine Serum Albumin [10mg/ml], 0.5µl forward and 0.5µl reverse primers [10µM], 0.3µl Invitrogen Taq DNA polymerase [5unit/µl], 1µl DNA template and 10.7µl water. The thermocycler protocol comprised a 4 min initial denaturation (at 94°C) followed by 40 cycles of 1 min denaturation (at 94°C), 1 min annealing (at 51°C for universal primers and 63°C for group specific primers) and 1.5 min elongation (at 72°C). Annealing temperature was elevated to 63°C to enhance group-specific primer specificity during the second (nested) PCR. Filter tips and negative controls were used throughout the nested PCR to control for false positives and avoid contamination.

PCR products were processed through electrophoresis on a 1.5% agarose gel, followed by a gel extraction of the banding patterns and DNA purification (Qiagen Qiaquick© PCR gel extraction kit). All PCR products were diluted to the same concentration of 0.5 ng µl^-1^ to provide an even contribution from each individual snail, should it be a big adult or a very small juvenile. PCR products were pooled to make a unique 0.5 ng µl^-1^ DNA sample following the manufacturer’s recommendation for the *Roche Genome Sequencer FLX System*.

### BLAST analyses

Short DNA sequences, or ‘reads’, obtained by 454-pyrosequencing were compared to our earthworm DNA library (containing 15 species) using the BLAST programme [48]. Only reads of expected length and containing at least one complete primer were kept. BLAST ‘hits’ were used to assign reads to one of the reference earthworm species and statistical significance was measured by the E-value provided by the BLAST programme. The E-value describes the random background ‘noise’ that exists for matches between sequences, with high significance reached for E-value < 1e-5. To assure conservative identification, reads were assigned to one reference species only when they had either a single hit with E-value < 1e-5 or multiple hits with the second best hit being clearly much worse than the top hit (i.e., top hit E-value / second hit E-value < 1e-5). Reads that did not fulfil these conditions were compared to the Genbank database using the BLASTn algorithm to confirm that they corresponded to earthworm DNA. If so, they were considered additional species (i.e., not present in the DNA library). The quality of DNA reads was measured using Phred [49] and only sequences with Phred score > 30 (i.e. base call accuracy > 99.9%) were used. Also, amplicons with unexpected lengths (<120 bp or >160 bp), amplicons lacking a complete primer and amplicons detected less than 5 times in total were considered as potential chimeric sequences or PCR artefacts and were discarded. The complete dataset has been deposited in a NCBI Bioproject (http://www.ncbi.nlm.nih.gov/bioproject) under the accession number PRJNA210725.

**Figure 2:**
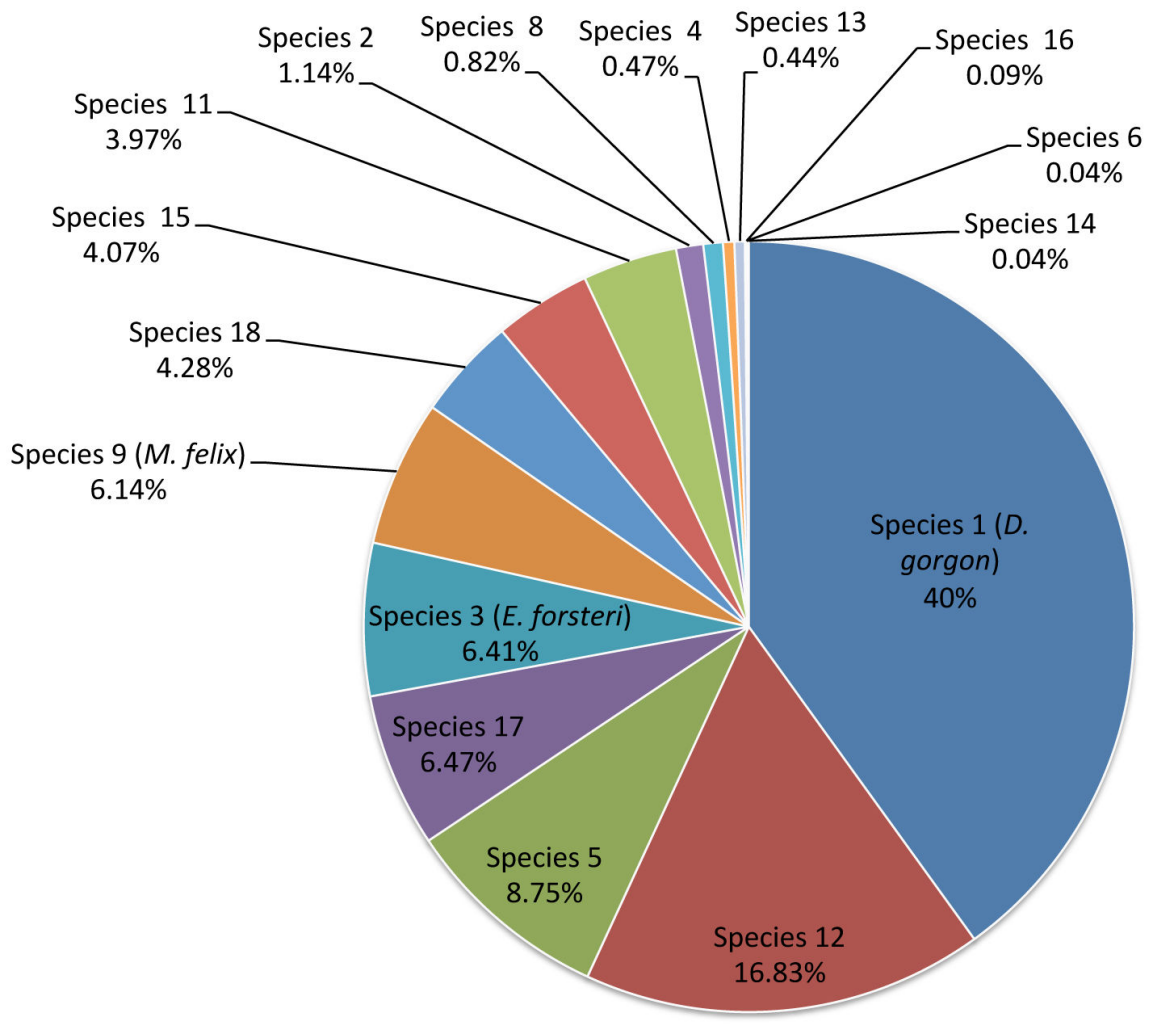
Breakdown of predated earthworm species. Proportion of the 8,712 good-quality amplicons from each of 16 different earthworm species based on amplicons obtained after 454-pyrosequencing on 46 snail faecal strings.

## Results

**Figure 3:**
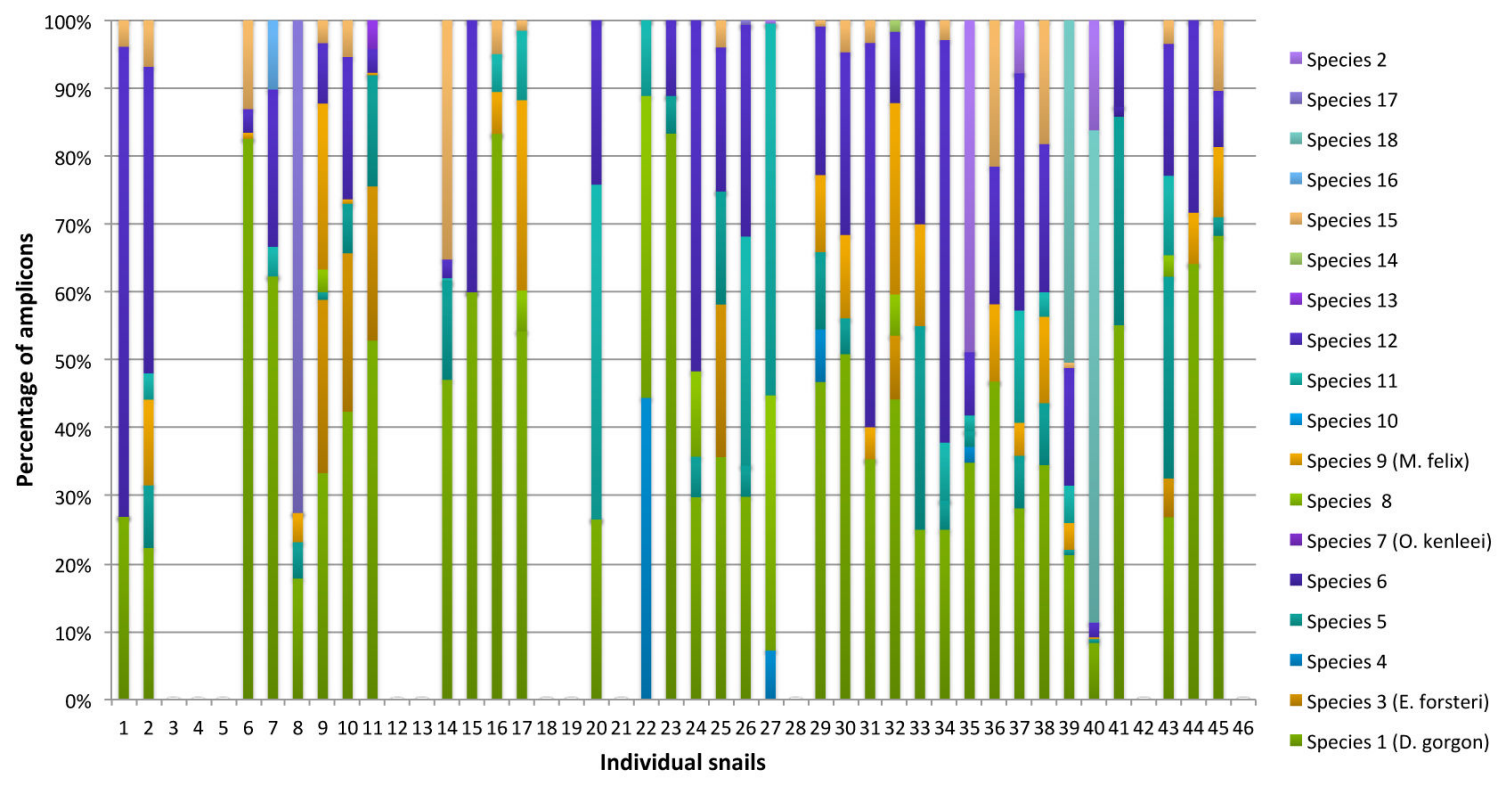
Individual diet composition for 46 *Powelliphantaaugusta* snails. Percentage of amplicons detected for each earthworm species in each faecal string (n=46).

**Figure 4:**
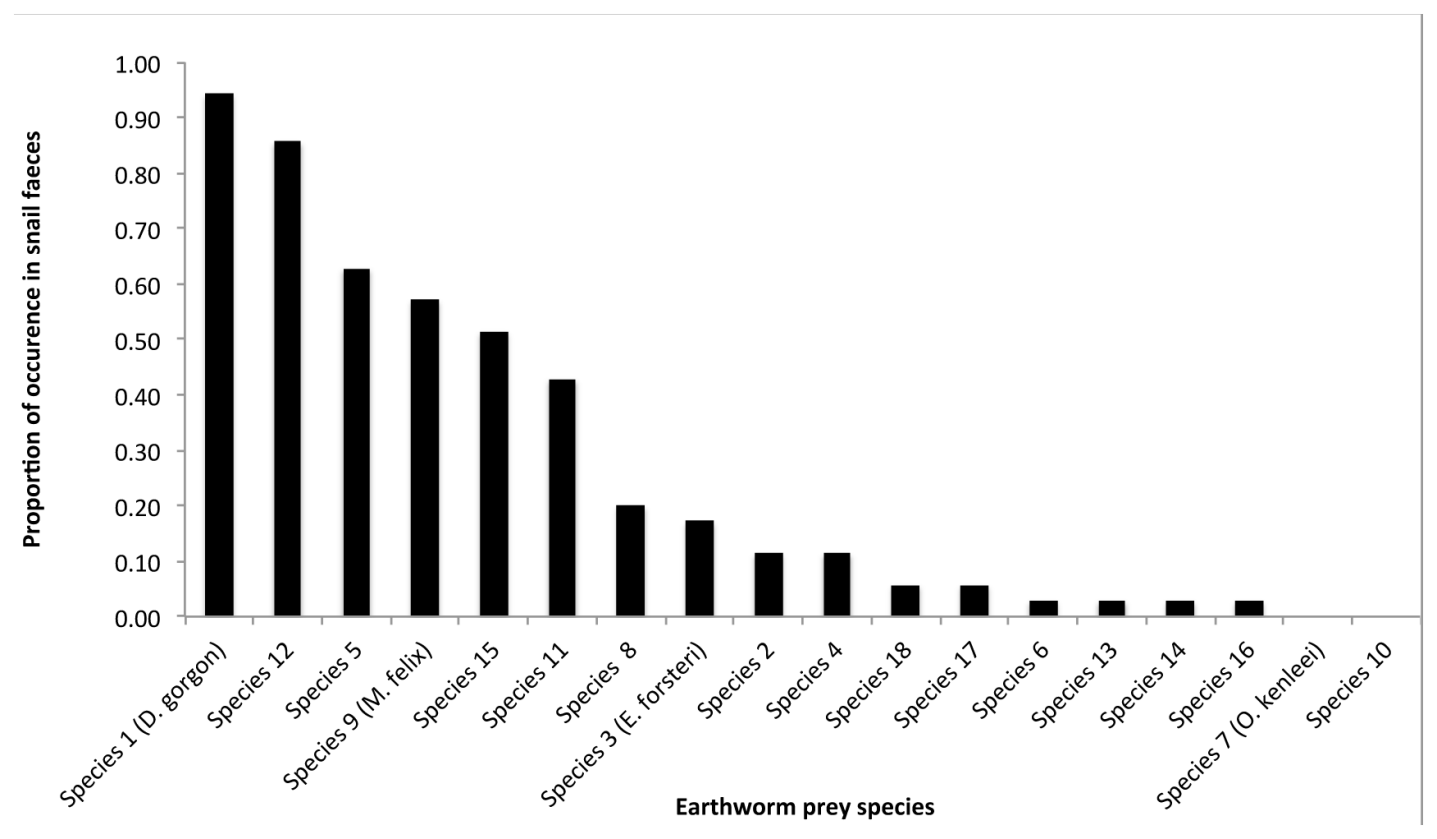
Frequency of occurrence of each species of earthworm in the diet of *P* *. augusta* . Proportion of faecal strings (n=35) that contained DNA from each earthworm species.

The pyrosequencing analysis revealed the presence of earthworm DNA in 35 of the 46 snail faecal samples. The proportion of samples where earthworm DNA was not detected was similar across age classes (15 to 22%) except for hatchlings where two samples out of three were negative. A total of 8,742 DNA fragments were successfully amplified and sequenced from the 35 faecal samples that contained earthworm DNA. BLAST analysis of all good-quality amplicons revealed that 7,120 (83%) of them matched with one of the species in the earthworm DNA library. Thirteen species from the library were detected while two of them (species 7 and 10) were absent from the faecal samples. The remaining amplicons (17%) corresponded to three additional taxa that were not present in the original earthworm library. Because divergence from the closest library species was greater than 7.5%, these taxa were considered as three additional species and noted species 16, species 17 and species 18 thereafter. The breakdown of amplicons per species of earthworm shows that one species in particular (species 1, *D* *. gorgon* ) was most frequent in the sequence data, with 40% of all amplified amplicons corresponding to that species (Figure 2). Most faecal samples contained DNA from several species of earthworms (Figure 3). The mean number of earthworm species present in one snail faecal string was 3.4 (± 0.4). No differences were observed in relation to snails’ age class for the number of species predated (ANOVA, F_4,30_ = 1.6843 p = 0.1796) or which species had been consumed (*F*-test, F_1,9_ = 0.790, p = 0.752). Species 1 ( *D* *. gorgon* ) and species 12 were predated by most snails, with their DNA present in 94% and 86% of the faecal strings respectively (Figure 4). Other highly-predated species were species 5 and species 9 ( *M* *. felix* ) found in 63%, 57% of the faecal strings, and to a lesser extent species 15 and 11 found in 51% and 43% of the faecal strings. Species 2, 3 ( *E* *. forsteri* ), 4, 8, 17 and 18 were occasionally predated (present in 6-20% of the faecal samples); species 6, 13, 14, and 16 were rarely predated (typically found in one faecal sample only) and species 7 ( *O* *. kenleei* ) and species 10 were not predated by any of the snails (Figure 4).

## Discussion

### Diet composition of *P* *. augusta*

The molecular diet analysis protocol used here allowed the amplification, sequencing and identification of 16 different species of earthworms comprising the diet of 35 *P* *. augusta* snails. Based on these samples, no ontogenetic shift was observed in the diet of the snail, however, sample size was very limited for the youngest age class. This mollusc appears to be able to feed on a wide variety of earthworm species since all but two of the species inventoried in their distribution area were detected in their faeces. This result partly supports the current use of the exotic earthworm *Eisenia fetida* Bouché [50] as a food source for the captive population of *P* *. augusta* (pers. obs.). An important limitation of molecular diet analysis is that, due to differences in prey size, it is difficult to quantify the relative contribution of each prey species in the diet [51]. The relative amounts of each amplicon do not necessarily provide an accurate quantification of the contribution of each predated species to the snails’ overall diet. Rather they merely reflect the amount of prey DNA, which can be influenced by the size of the predated earthworm (large species are likely to produce more amplicons), the time since consumption (earthworms eaten earlier are likely to produce fewer amplicons) and possible molecular bias in favor of certain species (i.e., some amplicons may be more prone to amplification than others). Quantitative PCR (qPCR) has been used to estimate the relative quantity of each prey species in faecal samples but this requires careful validation with strictly controlled feeding trials [51], which are often not achievable for the species of interest. As an alternative to qPCR, the use of molecular tags [45] allowed an estimation of the proportion of faeces containing DNA from a particular species of earthworm, which is a surrogate for the proportion of individual snails predating that species. This provided a good estimate of the importance of each prey species in the diet of these snails as a whole, and showed that snails of all ages had comparable diets, with certain species of earthworms consistently predated more than others. In a recent study, Murray et al. [52] indicated that 454-pyrosequencing provides a very similar estimation of prey quantities when compared to qPCR. However in our study, the proportion of amplicons obtained from 454-pyrosequencing appears as a poor estimate of the diet. For example, species 17, which was the fourth most important source of amplicons over all faecal samples (Figure 2), was only predated by two individual snails (Figure 4).

Because little is known about these earthworms, it is difficult to assess why certain species are predated more than others. As suggested in a previous paper these snails may indiscriminately eat earthworms as they are found rather than specifically foraging for particular species [15]. In this case, the main drivers of earthworm predation are likely to be the relative abundance of each earthworm species, and their ecological guild, i.e. position in the soil profile. Typically, epigeic earthworm species (living in the leaf litter) and anecic species (living in deep burrows, but foraging in the litter at night) would be more likely to be predated than would endogeic ones (living almost exclusively in the soil) [50]. A quantitative assessment of earthworm populations would allow the comparison between earthworm abundance and predation. However, as mentioned by other authors, such assessment is difficult to produce because of biases in earthworm sampling methods, difficulty in identifying juveniles and patchy distribution of earthworms in heterogeneous habitats [53]. In our case, it was made even more difficult because most earthworm species were undescribed and more importantly most of the snail original habitat had been lost to coal mining. There is therefore little information available about the abundance of these earthworm species; however, some ecological data exist for four species included in the inventory. *O* *. kenleei* (species 7) was described as endogeic [41] and was not detected in any of the analysed snail faecal samples. *D* *. gorgon* (species 1) and *M* *. felix* (species 9) were both described as anecic [41] and these were found in 94% and 51% of the faecal samples, respectively. *E* *. forsteri* , described as epigeic [40], was found in 6% of the faecal samples. This seems to support the hypothesis that snails forage randomly through the litter and predate any earthworm they encounter. 

### Application of NGS to diet analyses

Next-generation sequencing provides a good compromise in terms of precision in species identification, information obtained (DNA sequences), and prompt delivery of results. In addition, many samples can be analysed simultaneously for little additional cost. The use of molecular tags allows mixing of samples from different origins in a single reaction [45]. With decreasing costs, NGS is destined to become a standard tool for the study of endangered predatory species that are difficult to observe directly. However, the diet analysis protocol used here demands a precise framework to be applied:

(1) A comprehensive inventory of the potential prey species is required, at least at the level of higher taxonomic ranks, to design appropriate group-specific primers. Unexpected prey can be detected and identified *a posteriori* if the group-specific primers are general enough to amplify their DNA.

(2) Because many NGS systems are currently limited in the length of the fragments they can process, amplicons must be short (often <250bp). Although, on-going improvement of NGS technologies (e.g. 454 FLX+, Ion PGM) as well as the development of new sequencing platforms (e.g. PacBios RS) may rapidly overcome this size limitation [54], short amplicons will remain a constraint for degraded DNA.

(3) The choice of a small target fragment or “mini-barcode” is crucial. It must be short enough for compatibility with NGS and degraded DNA, but informative enough to ensure accurate species identification. We recommend using objective tools such as the sliding window function in the R package SPIDER [42] to optimise identification performance with short amplicons.

## Conclusion

Our study reveals that *P* *. augusta* can predate a broad range of earthworm species and probably feeds on any species of earthworm that is available to them. The variety of prey reflects the diversity of earthworm species present in the Stockton plateau. Such non-specificity has important implications for the conservation management of *P* *. augusta* . The choice of new relocation areas, the feeding protocol for captive snails, and the ecological restoration of their original habitat are simplified because a range of earthworm communities seems to be suitable for providing prey to snails in all these conditions. More generally, the development of such methods has major implications for the conservation of endangered invertebrate and vertebrate species whose dietary requirements are unknown. This represents a significant improvement compared to traditional methods used in the field of molecular analyses of predation [14].
