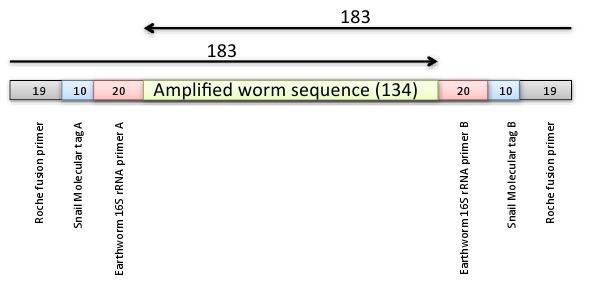# Correction: Using Next-Generation Sequencing to Analyse the Diet of a Highly Endangered Land Snail (*Powelliphanta augusta*) Feeding on Endemic Earthworms

**DOI:** 10.1371/annotation/9606710c-bae7-485d-a656-3a42c2c77d85

**Published:** 2013-10-15

**Authors:** Stéphane Boyer, Stephen D. Wratten, Andrew Holyoake, Jawad Abdelkrim, Robert H. Cruickshank

The version of Figure 1 that appeared in the paper was incomplete. A complete and correct version of the the figure is available here: 

**Figure:**